# Impaired Facial Emotion Recognition in Individuals at Ultra-High Risk for Psychosis and Associations With Schizotypy and Paranoia Level

**DOI:** 10.3389/fpsyt.2020.00577

**Published:** 2020-06-26

**Authors:** Eunchong Seo, Hye Yoon Park, Kyungmee Park, Se Jun Koo, Su Young Lee, Jee Eun Min, Eun Lee, Suk Kyoon An

**Affiliations:** ^1^Section of Self, Affect and Neuroscience, Institute of Behavioral Science in Medicine, Yonsei University College of Medicine, Seoul, South Korea; ^2^Department of Psychiatry, Yonsei University College of Medicine, Severance Hospital, Seoul, South Korea; ^3^Department of Hospital Medicine, Yonsei University College of Medicine, Yongin Severance Hospital, Gyeonggi-do, South Korea; ^4^Graduate Program in Cognitive Science, Yonsei University, Seoul, South Korea; ^5^Department of Psychiatry, Myongji Hospital, Gyeonggi-do, South Korea

**Keywords:** facial emotion recognition, inaccuracy, negative response bias, schizotypy, paranoia, ultra-high risk for psychosis, schizophrenia

## Abstract

**Background:**

Patients with schizophrenia and individuals at ultra-high risk for psychosis (UHR) have been reported to exhibit impaired recognition of facial emotion expressions. This impairment has involved both inaccuracy and negative bias of facial emotion recognition. The present study aimed to investigate whether UHR individuals display both types of impaired facial emotion recognition and to explore correlations between these impairments and schizotypy, as well as paranoia levels, in these individuals.

**Methods:**

A total of 43 UHR individuals and 57 healthy controls (HC) completed a facial emotion recognition task consisting of 60 standardized facial photographs. To explore correlations, we assessed schizotypy using the Revised Physical Anhedonia Scale and Magical Ideation Scale and paranoia level using the Paranoia Scale and persecution/suspicious item of the Positive and Negative Syndrome Scale in UHR individuals.

**Results:**

Compared with HC, UHR individuals exhibited less accuracy for facial emotion recognition (70.6% vs. 75.6%, p=0.010) and a higher rate of “fear” responses for neutral faces (14.5% vs. 6.0%, p=0.003). In UHR individuals, inaccuracy was significantly correlated with schizotypy scores, but not with paranoia level. Conversely, “disgust” response for neutral faces was the only fear response correlated with paranoia level, and no threat-related emotion response correlated with schizotypy scores.

**Discussion:**

UHR individuals exhibited inaccuracy and negative bias of facial emotion recognition. Furthermore, schizotypy scores were associated with inaccuracy but not with negative bias of facial emotion recognition. Paranoia level was correlated with “disgust” responses for neutral faces but not with inaccuracy. These findings suggest that inaccuracy and negative bias of facial emotion recognition reflect different underlying processes, and that inaccuracy may be a vulnerability marker for schizophrenia.

## Introduction

Patients with schizophrenia exhibit deficits in social cognition that produce difficulties in social interactions (1). Social cognition consists of various psychological processes involved with recognizing the mental state of other people (2, 3). Facial emotion recognition—the ability to evaluate another person’s emotional state from their facial expressions—is one of the most studied social cognition processes in schizophrenia (3, 4). Impaired facial emotion recognition has been repeatedly observed in previous studies of patients with first-episode schizophrenia, as well as chronic schizophrenia (4–6). Thus, impaired facial emotion recognition could represent a trait marker of psychotic disorders. This premise is supported by findings of impaired facial emotion recognition in individuals at ultra-high risk for psychosis (UHR) (7–10).

Two types of impaired facial emotion recognition have been previously reported in patients with diagnosed schizophrenia, as well as in UHR individuals: inaccuracy and negative bias. Inaccuracy, which implies a lack of ability to accurately recognize facial emotions, was a consistent finding in most previous studies of schizophrenia (4, 11) and UHR (8, 12, 13). Negatively biased error patterns for neutral faces was also observed in previous studies of schizophrenia (14–16) and UHR (8). Although the specific emotion categories that were biased differed according to the characteristics of the research subjects and emotion recognition tasks, most studies showed bias toward negative emotions, such as “disgust” (14), “anger” (15, 16), and “fear” (16) in patients with schizophrenia and bias toward “anger” in UHR individuals (8). These emotions (fear, anger, disgust) are interrelated, threat-related emotions. Fear and anger are well known facial emotions associated with social threats (17, 18). With disgust, the type of threat is different, but it is similar to fear (warning others of the presence of danger) and anger (displaying anger toward others) in that it is a defensive emotion about a possible threat (with disgust toward others representing a type of contamination fear). Also, in the sense that disgust represents the rejection of a stimulus, disgust could appear as a rejection to others to avoid. In this respect, disgust could be thought of as a similar group of emotions that can be perceived as being hostile such as fear and anger in paranoia. (19–21). Although bias toward threat-related emotions seem to be consistent in schizophrenia (14–16), few studies have evaluated this bias in the UHR phase (8). Thus, it remains unclear whether negatively biased error patterns represent a trait marker that is already apparent during the putative “prodromal” UHR period.

The relationship between facial emotion recognition and psychometrically-identified schizotypy has also been studied because of the possibility of impaired facial emotion recognition as a vulnerability marker for psychosis. Relatively recent studies consistently showed lower accuracy of facial emotion recognition in individuals with high degrees of schizotypy (22–26). In addition, some studies have reported an association between negative bias of facial emotion recognition and schizotypal features in the general population. (23, 24) However, since most previous schizotypy studies have been conducted in general populations (23–25, 27, 28), the relationship between facial emotion recognition and schizotypy has not been studied sufficiently in clinical populations. Our previous study (10) of UHR individuals and patients with first-episode schizophrenia showed a significant correlation between inaccuracy of facial emotion recognition and schizotypy, but the relationship between negative bias of facial emotion recognition and schizotypy has not yet been examined. As the presence of schizotypy has been suggested to confer proneness to schizophrenia spectrum disorders (29), associations between schizotypy and facial emotion recognition suggest that impaired facial emotion recognition could be a vulnerability marker of psychosis. Therefore, exploration of the association between the two types of impaired facial emotion recognition (inaccuracy and negative bias) and schizotypy in clinical populations, such as UHR individuals, would be helpful for assessing whether each type of facial emotion recognition impairment is a potential vulnerability marker for schizophrenia spectrum disorders.

Furthermore, actively paranoid patients with schizophrenia were reported to exhibit no difference in accuracy of facial emotion recognition but tended to be more likely to judge a neutral face as “anger”, when compared with non-paranoid patients (15). These findings suggest that paranoia level may be related to negative bias, but not inaccuracy, of facial emotion recognition. The association between paranoia and negative bias towards threat-related emotions is consistent with the existing hypothesis that paranoid patients tend to be more aware of threats in ambiguous situations (17, 18). This hypothesis was supported by recent studies showing that schizophrenia patients take longer to process ambiguous stimuli for some negative emotions (sad, anger) (30), and reduced visual scanning of salient features mediate paranoia and facial emotion recognition (31). These findings may be one of possible mechanisms explaining the correlation between paranoia and negative bias of facial emotion recognition that we expect. Together with the consistent prior reports of facial emotion recognition inaccuracy in patients with schizophrenia, it can be hypothesized that accuracy of facial emotion recognition is related to inherent traits of schizophrenia, whereas negative bias of facial emotion recognition is related to paranoia level, not to inherent traits of schizophrenia.

Based on these previously reported findings, we conducted a study of UHR individuals to test the following three hypotheses: 1) UHR individuals exhibit inaccuracy and negative bias of facial emotion recognition; 2) schizotypy in UHR individuals is associated with inaccuracy and negative bias of facial emotion recognition; and 3) paranoia level of UHR individuals is unrelated to inaccuracy of facial emotion recognition but does correlate with negative bias towards threat-related emotions (anger, fear, and disgust). In addition, it was explored whether there were emotion specific deficits in UHR individuals.

## Methods

### Participants

A total of 43 UHR individuals and 57 healthy controls (HC) were enrolled in this study between April 2008 and December 2011. Some of the participants overlap with existing our prior study examining the accuracy of facial emotion recognition using different facial photos from Japanese and Caucasian Facial Expressions of Emotion and Neutral Faces (32). All participants were evaluated using the Structured Clinical Interview for DSM-IV (33, 34). According to the Criteria of the Prodromal Syndromes from the Structured Interview for Prodromal Syndromes (35), UHR individuals were defined as people who met the criteria for at least one of these three prodromal syndromes: (1) brief intermittent psychotic syndrome; (2) attenuated positive prodromal syndrome; and (3) genetic risk and deterioration syndrome. The study protocol was approved by the Institutional Review Board of Severance Hospital. Written informed consent was obtained from all participants after being provided with a full explanation of the study’s procedures. For participants under the age of 18 years, we also obtained informed consent from their parents. Demographic and clinical profiles of the participants are summarized in **Table 1**.

**Table 1:** Demographic and clinical profiles of healthy controls and individuals at ultra-high risk for psychosis.

	Healthy controls (n = 57)	UHR individuals (n = 43)	P-value
Age (years)	20.9 (3.3)	19.9 (3.6)	0.937
Education (years)	13.3 (1.9)	12.8 (2.0)	0.819
Sex (male/female)	33/24	25/18	0.980
SIPS-defined prodromal status (BIPS/APS/GRDS)	–	8/39/7	–
PANSS, positive scale^1^	–	13.8 (3.9)	–
PANSS, negative scale^1^	–	16.1 (5.1)	–
PANSS, general psychopathology scale^1^	–	32.0 (7.9)	–
Revised Physical Anhedonia Scale	–	23.7 (9.8)	–
Magical Ideation Scale	–	10.6 (5.9)	–
Suspiciousness/persecution item of PANSS^1^	–	2.83 (1.0)	–
Paranoia Scale^2^	–	36.0 (18.2)	–
Antipsychotic medication	–	27/16	–
Naïve/medicated			
Chlorpromazine equivalent dose (mg/day)*	–	133.6 (77.9)	–

Data are mean (standard deviation) or number.

^1^Data missing for one person.

^2^Data missing for three people.

*Kroken et al. (36).

APS, Attenuated Positive Symptom Prodromal Syndrome; BIPS, Brief Intermittent Psychotic Symptom Prodromal Syndrome; GRDS, Genetic Risk and Deterioration Prodromal Syndrome; SIPS, Structured Interview for Prodromal Syndromes. (35); UHR, Ultra-High Risk for psychosis.

PANSS, Positive and Negative Syndrome Scale (37); Revised Physical Anhedonia scale (38); Magical Ideation Scale (39); Paranoia Scale (40).

### Procedures

The facial emotion recognition task consisted of 55 facial photographs selected from standardized photographs of the Ekman and Friesen series (41). We selected those photographs for which consensus was reached by more than 70% of observers in our previous study of 134 Korean youths (42). The photographs represented six different emotions, as well as neutral faces: 10 showed happiness, 6 showed disgust, 6 showed anger, 9 showed sadness, 10 showed surprise, 4 showed fear, and 10 showed neutral expressions. The study participants were shown each photograph in a pseudorandom order and asked to choose an emotion category that most appropriately described the emotional state of the person in the photograph. The category options were happiness, disgust, anger, sadness, surprise, or fear, which were typed below each facial photograph. While 9 photographs of neutral faces were included, neutral was not included as a response category. The accuracy rate for recognizing neutral faces is known to be very high, and we were concerned that if neutral were included as a response option, we would be unable to analyze tendencies to attribute each emotion to neutral faces because of a limited number of misattribution cases. The stimulus presentation time was 7 seconds on computer screen and then the labels of six emotional categories were displayed on the screen for another 7 seconds for response time. During the response time, participants were allowed for choosing the emotional category in response paper sheet.

Schizotypy was assessed using the Revised Physical Anhedonia Scale (38) and the Magical Ideation Scale (39). The Revised Physical Anhedonia Scale is a self-reported scale consisting of 61 items that assess deficits in the ability to derive pleasure from typically pleasurable physical stimuli, such as sex and food. It has been used widely in schizotypy research in both clinical and non-clinical settings (43) and has exhibited fair reliability and internal consistency (44). The internal consistency (Cronbach’s alpha) of the Revised Physical Anhedonia Scale in the present study was 0.66. The Magical Ideation Scale is a self-reported questionnaire with 30 items that assess magical thinking. The developers of this scale defined magical thinking as “the tendency to accept forms of causality that are not viewed as valid in our culture” (45). This scale has demonstrated good reliability and internal consistency in previous studies (46, 47). The internal consistency (Cronbach’s alpha) of the Magical Ideation Scale in the present study was 0.77.

Paranoia level was assessed using the Paranoia Scale (40) and the persecution/suspicious item of the Positive and Negative Syndrome Scale [PANSS; (37)]. The Paranoia Scale is a self-reported assessment of paranoid ideation, which consists of 20 items. Both paranoia level scales have been reported to have good psychometric properties and have been widely used in research involving paranoia in clinical and non-clinical settings (48, 49). The Korean versions of the paranoia level scales have also shown acceptable validity and reliability and have been widely used in Korean research (50, 51). The internal consistency (Cronbach’s alpha) of Paranoia scale in the present study was 0.95.

The clinical interviews and assessments of psychopathology, including PANSS, were administered by a psychiatrist on the day of enrollment in the study. Each participant then completed the Paranoia Scale, Revised Physical Anhedonia Scale, and Magical Ideation Scale. The facial emotion recognition task was conducted by a masters-level psychologist within 1 week of enrollment.

### Data Analysis

Performance during the facial emotion recognition task was quantified using two indices. First, total hit rate was used to measure accuracy. As previously mentioned, since “neutral” was not provided as the answer, the total hit rate was calculated excluding the response to the neutral stimuli. To compare the total accuracy rate of the facial emotion recognition task between UHR individuals and HC, we used the independent-sample t-test.

To measure negative bias, the response rate of specific emotions for neutral faces was calculated. If the proportion of negative emotions in the reaction to the neutral faces is high, there is a negative bias. To examine differences between response rates for neutral faces between groups, we performed multivariate analysis of variance.

For the exploratory analysis of specific deficits in emotion category in UHR individuals, the independent-sample t-test was performed to compare the accuracy of each specific emotion category between UHR individuals and HC.

Correlations between the total accuracy rate on the facial emotion recognition task and the values on both scales of paranoia level, as well as schizotypy, were examined using Pearson’s correlation analysis. To examine the relationships between threat-related emotion response rates for neutral faces and the values on both scales of paranoia level, as well as schizotypy, we used Spearman’s correlation analysis because we assumed that these threat-related emotion responses may not follow a normal distribution.

In all correlation analyses, we used the Bonferroni correction to adjust p-values. The significance level was set at 0.05 for all tests.

## Results

### Facial Emotion Recognition Task Performance

With regards to accuracy, facial emotion recognition task performance was worse in UHR individuals (mean=80.0%, standard deviation [SD]=12.7) than in HC. (mean=85.7%, SD=8.8; t=2.65; p=0.009).

Regarding the negative bias, in emotion-specific responses to neutral faces, there was statistically significant difference between the NC and UHR individuals. (Wilks’ Lambda=0.89, F (5,94)=2.32, p=0.049). Specifically, the “fear” response rate was significantly higher for UHR individuals (mean=14.2%, SD=14.2) than for HC (mean=6.1%, SD=12.1; p=0.003). Response rates for the other threat-related emotions (“anger” and “disgust”) were not significantly different between UHR individuals and HC. Response rates for other emotions (“happiness”, “sadness”, and “surprise”) also did not differ significantly between UHR individuals and HC. Details of these results are shown in **Table 2**. In exploratory analysis of the difference in accuracy for each emotion category, UHR individuals (mean=69.8%, SD=23.5) show significantly lower accuracy rate of sad emotion than HC (mean=83.6, SD=12.8; p<0.001). Details of each category are shown in **Supplementary Material**.

**Table 2:** Response rates for neutral faces in healthy controls and individuals at ultra-high risk for psychosis.

	Heathy controls (n=57)	UHR individuals (n=43)	P-value
Responses for neutral faces			
Happiness	16.8 (23.2)	14.2 (22.3)	0.566
Sadness	38.9 (24.9)	31.4 (22.4)	0.121
Surprise	6.5 (9.5)	7.4 (10.5)	0.638
Disgust	4.9 (13.9)	6.2 (12.9)	0.617
Anger	26.7 (17.5)	26.5 (20.0)	0.967
Fear	6.1 (12.1)	14.2 (14.2)	0.003*

*p < 0.05. Data are number (percentage).

UHR, individuals at Ultra-High Risk for psychosis.

### Correlation Between Total Accuracy Rate and Schizotypy Scores, as Well as Paranoia Level, in UHR Individuals

**Figure 1:**
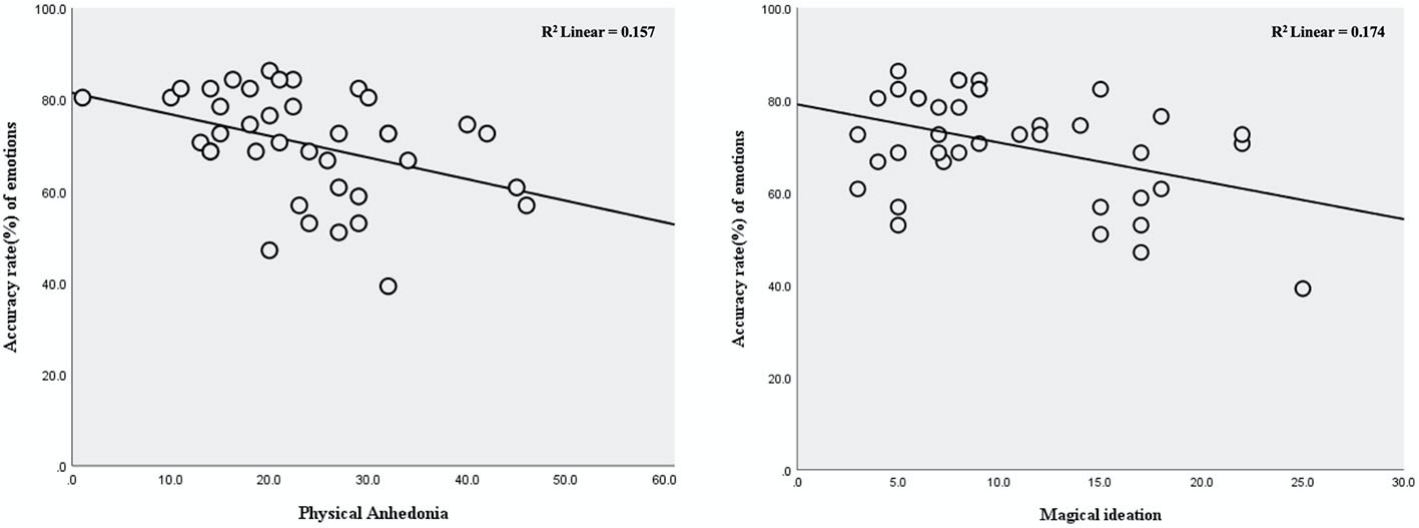
Relationships between accuracy rate for facial emotion recognition and schizotypy scores in individuals at ultra-high risk for psychosis.

Total accuracy rate was significantly correlated with schizotypy scores on both schizotypy scales: Revised Physical Anhedonia Scale: r = −0.396, corrected p<0.050, and Magical Ideation Scale: r = −0.417, corrected p=0.033 (**Figure 1**, **Table 3**). Total accuracy rate was not significantly correlated with paranoia level determined by either the Paranoia Scale or the suspiciousness/persecution item of the PANSS (p>0.129 for both).

**Table 3:** Correlation coefficients for the associations between accuracy rate of facial emotion recognition/threat-related emotion response rates for neutral faces and schizotypy scores and paranoia level in individuals at ultra-high risk for psychosis.

		Schizotypy score		Paranoid level	
		Revised Physical Anhedonia Scale (n=39)	Magical Ideation Scale (n=39)	Paranoia Scale (n=40)	Suspiciousness/persecution item of PANSS (n=42)
Accuracy rate		−0.396 (corrected P<0.050)*	−0.417 (corrected P=0.033)*	−0.344 (corrected P=0.129)	−0.135 (corrected P>0.999)
Response for neutral faces	“Disgust” response	0.246 (corrected P>0.999)	0.330 (corrected P=0.470)	0.501 (corrected P=0.012)*	0.449 (corrected P=0.032)*
	“Angry” response	0.004 (corrected P>0.999)	0.193 (corrected P>0.999)	−0.043 (corrected P>0.999)	−0.057 (corrected P>0.999)
	“Fear” response	0.105 (corrected P>0.999)	−0.047 (corrected P>0.999)	0.164 (corrected P>0.999	0.108 (corrected P>0.999)

*Bonferroni-corrected P < 0.05 (for accuracy rate: uncorrected P<0.05/4; for response for neutral faces: uncorrected P<0.05/12).

PANSS, Positive and Negative Syndrome Scale (37); Revised Physical Anhedonia scale (38); Magical Ideation Scale (39); Paranoia Scale (40).

### Correlation Between Threat-Related Emotion Response Rates and Schizotypy Scores, as Well as Paranoia Level, in UHR Individuals

**Figure 2:**
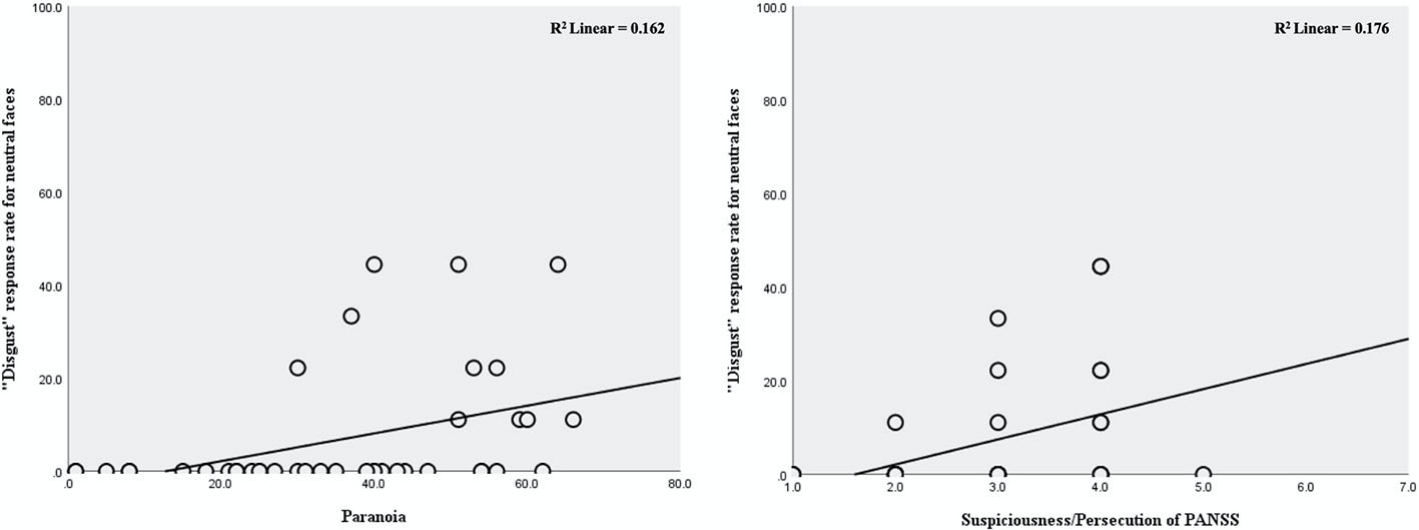
Relationships between “disgust” response rate for neutral faces and paranoia level in individuals at ultra-high risk for psychosis.

The response rate for “disgust” was significantly correlated with paranoia level determined by both the Paranoia Scale (r=0.501, corrected p=0.012) and the suspicious/persecution item of the PANSS (r=0.449, corrected p=0.032). There were no other significant correlations between threat-related emotion response rates and paranoia level. There were also no significant correlations between threat-related emotion response rates and schizotypy scores on either schizotypy scale (p>0.470 for all comparisons) (**Figure 2**, **Table 3**).

## Discussion

### Results and Comparisons to Previous Studies

To our knowledge, this is the first published study examining the associations between two types of impaired facial emotion recognition (inaccuracy and negative bias) and schizotypy, as well as paranoia level, in UHR individuals. In the present study, UHR individuals exhibited both types of impaired facial emotion recognition: inaccuracy and negative (“fear”) bias. Moreover, inaccuracy of facial emotion recognition was correlated with schizotypy scores, whereas the “disgust” response rate for neutral faces was correlated with paranoia level.

In the present study, UHR individuals had lower total accuracy for facial emotion recognition (70.6% vs. 75.6%) and higher rates of “fear” responses to neutral faces (14.5% vs. 6.0%), when compared with HC. This inaccuracy was consistent with the results of previous studies in UHR individuals (10, 13, 52). The negative bias toward “fear” emotion was also generally consistent with the results of previously studies involving UHR people (8) and patients with schizophrenia (14–16, 21), although “anger” was the emotion that was biased in the previous UHR study. The difference between the specific biased emotion categories may arise from methodological differences between studies, such as differences in the types of facial emotional stimuli or the type or number of emotion categories. The effect of methodological differences has already been demonstrated in previous schizophrenia studies showing negative bias for different emotion categories, such as “disgust”, “fear”, and “anger” (14–16, 21). Considering methodological differences, the “fear” bias observed in the present study is consistent with the “anger” bias of the previous UHR study (8), as both represent biases for threat-related emotions. As with facial emotion recognition inaccuracy, threat-related emotion bias is observed in both UHR individuals and patients with schizophrenia, suggesting that threat-related emotion recognition bias may represent a marker of psychosis. To provide support for this suggestion, further studies addressing methodological issues would be helpful.

We also explored relationships between the two types of impaired facial emotion recognition and schizotypy, as well as paranoia level. Schizotypy scores correlated with inaccuracy of facial emotion recognition, but not with the response rates for any of the three threat-related emotions. The correlation between inaccuracy and schizotypy is consistent with the results of our previous study of UHR individuals and patients with first-episode schizophrenia (10), as well as previous general population studies (23, 25, 26). However, the lack of negative bias observed in this study contrasted with the results of previous studies in the general population, which showed correlations between high schizotypy and negative bias (23, 24). This discrepancy suggests that although both inaccuracy and negative bias of facial emotion recognition exist in the putative “prodromal” UHR phase, the processes underlying these impairments may differ. Considering the characteristics of schizotypy, which reflects proneness to psychosis (29), the significant association between inaccuracy and schizotypy scores suggests that inaccuracy is likely a vulnerability factor for schizophrenia spectrum disorders.

When examining paranoia, we found that inaccuracy was not associated with paranoia level and that the “disgust” response was the only threat-related emotion correlated with paranoia level in UHR individuals. The lack of association between paranoia level and inaccuracy is consistent with the results of a previous study, which reported no difference in accuracy of facial emotion recognition between patients with paranoid or non-paranoid schizophrenia (15). Unlike schizotypy, paranoia does not appear to be an inherent trait of psychosis but a symptom that changes according to the severity of the psychotic disorder. Thus, our finding that inaccuracy correlates only with schizotypy scores and not with paranoia level further supports the possibility that inaccuracy of facial emotion recognition is a trait marker for psychosis.

Although the “disgust” response rate correlated with paranoia level, which may correspond with the previous finding of more “anger” bias in patients with paranoid schizophrenia than in those with non-paranoid schizophrenia (15), we cannot definitively conclude that negative bias of facial emotion recognition correlates with paranoia level based on these findings. Pinkham et al. examined differences between paranoid and non-paranoid patients but did not evaluate the correlation between paranoia level and negative bias. In the present study, correlation between paranoia level and threat-related emotion (“disgust”) response was observed, but this emotion differed from the emotion that was biased in our UHR group (“fear”), compared with HC. One possible explanation for this discrepancy could be that paranoia is necessary for negative bias, but the level of paranoia is not directly proportional to negative bias. Alternatively, paranoia level may actually correlate with negative bias, but we were unable to detect this correlation because of the characteristics of UHR individuals. As these individuals exhibit less severe psychiatric symptoms, including paranoia levels, than those with schizophrenia, impaired facial emotion recognition is also likely to be less severe in UHR individuals. Thus, there is a possibility that the negative bias, paranoia level, or both were not of sufficient severity to permit detection of a significant correlation. In addition, UHR is an extremely heterogeneous group, with varying outcomes on follow-up: some individuals will proceed to develop a schizophrenia spectrum disorder, some will recover, and others will maintain their current status. If the negative bias is observed in only specific subgroups of patients with schizophrenia who have paranoid features, then any correlation effects would be further diluted by mixing paranoid-prone individuals with other people in the heterogeneous UHR group. Future studies examining subgroups of UHR individuals and patients with schizophrenia may help clarify these issues.

Regarding the exploration of specific deficits in emotion category in UHR individuals, there were lower accuracy rate of sad emotion significantly and those of happy and fear emotions in trend-level. These findings were globally compatible of the previous reports (9, 13). In near future, further study to clarify whether there is the emotion-specific deficits in UHR individuals under the application of the differential deficits design (53).

### Limitations

One limitation of the present study is that the ethnicity of people in the facial photographs differed from that of our Korean study participants. This difference could affect our results because racial and cultural differences may influence the interpretation of facial expressions. However, this effect was likely minimal because we used only those photographs with more than 70% consensus in our previous study of Koreans (42). Secondly, limitation may be that different numbers of stimuli were used for each emotion category to use only pictures with high inter-rater agreement. Thus, in the present study, the inaccuracy of facial emotion recognition was tested using the overall accuracy rates of facial photos across emotional categories. Since there was limitation of controlling the possible confounding effects due to different number of stimuli according the emotional category, it was only explored whether there were differential deficits according to the emotional category in UHR individuals. Meanwhile, in the case of negative bias, since we analyzed only the response to neutral stimuli, the effect of the difference in stimuli numbers would have been minimal. Thirdly, there may be at least partial relations of inaccuracy rates and negative bias of facial emotion recognition. In the presence of negative bias, the accuracy for positive or neutral stimuli may decrease, but the accuracy for negative stimuli may increase. Also, although the overall accuracy is not compromised, it may only show negative bias for stimuli that are generally difficult to match. In the opposite case, even if there is no bias, the ability to recognize facial emotions itself may be impaired. In this regard, possible relations of overall inaccuracy rate across emotional faces and negative bias to neutral ones may be small enough to be ignored in the present study. The difference in correlation with schizotypy and paranoid level, which shown in this study, also suggests that inaccuracy and negative bias are of different natures. Last potential limitation is that we used neutral face photographs as facial stimuli but excluded “neutral” as an emotion response option. We did this because we anticipated difficulties with measuring misattribution cases because of the very high accuracy for neutral faces observed in previous studies. However, this decision contributed to methodological differences between our current study and previous reports.

## Conclusions

In summary, UHR individuals exhibited impaired facial emotion recognition, including inaccuracy and negative bias. Schizotypy was associated with inaccuracy but not with negative bias of facial emotion recognition, whereas paranoia level was correlated with “disgust” response bias for neutral faces but not with inaccuracy. These findings suggest that there is a difference in the processes underlying the two types of facial emotion recognition impairments. Inaccuracy of facial emotion recognition may be a vulnerability marker for schizophrenia. To clarify the exact nature of negative bias of facial emotion recognition with respect to paranoia level, further investigations involving UHR individuals, as well as patients with schizophrenia, may be helpful.

## Data Availability Statement

The datasets generated for this study are available on request to the corresponding author.

## Ethics Statement

The studies involving human participants were reviewed and approved by Institutional Review boards at Severance Hospital. Written informed consent to participate in this study was provided by the participants’ legal guardian/next of kin.

## Author Contributions

SA designed the study. SA and EL recruited subjects. ES undertook the statistical analysis and wrote the first draft of the manuscript. ES, HP, KP, SK, SL, and JM interviewed patients and collected data. All authors contributed to the article and approved the submitted version.

## Conflict of Interest

The authors declare that the research was conducted in the absence of any commercial or financial relationships that could be construed as a potential conflict of interest.
