# Supplementary material for: Impaired Facial Emotion Recognition in Individuals at Ultra-High Risk for Psychosis and Associations With Schizotypy and Paranoia Level
Source: Front Psychiatry. 2020 Jun 26;11:577. doi: 10.3389/fpsyt.2020.00577 (PMC7333645; doi:10.3389/fpsyt.2020.00577)
Supplement: Supplementary file 1 [file Table_1.docx]

Supplementary table. Accuracy rates of facial emotion recognition in healthy controls and individuals at ultra-high risk for psychosis

|  | Healthy controls  (n=57) | UHR individuals  (n=43) | t | P-value |
| --- | --- | --- | --- | --- |
| Accuracy rate  for each emotion category |  |  |  |  |
| Happiness | 97.9 (7.5) | 94.0(13.8) | 1.83 | 0.07 |
| Sadness | 83.6 (12.8) | 69.8 (23.5) | 3.77 | <0.001 |
| Surprise | 90.0 (13.6) | 90.23 (13.5) | -0.09 | 0.93 |
| Disgust | 79.5 (25.2) | 75.2 (28.5) | 0.79 | 0.43 |
| Anger | 81.9 (18.4) | 78.7 (21.0) | 0.79 | 0.43 |
| Fear | 64.5 (30.2) | 51.7 (35.5) | 1.89 | 0.06 |

Data are number (percentage).

UHR: individuals at ultra-high risk for psychosis.
